# Comparing Sociodemographic, Health Status and Resources, Macroeconomic Status, and Environmental Factors on Infant Mortality Rates in Bahrain, Kuwait, and Oman: Longitudinal Time-Series Study

**DOI:** 10.2196/73203

**Published:** 2025-10-10

**Authors:** Mallak Yasir Al-Saqry, Rawaa Abubakr Abuelgassium Eltayib, Al-Hasan Ahmed Al-Busaidi, Moon Fai Chan

**Affiliations:** 1 Department of Family Medicine and Public Health, College of Medicine and Health Science, Sultan Qaboos University, Muscat, 123, Oman, 968 24141132

**Keywords:** infant mortality rate, sociodemographic, health status and resources, macroeconomic, environment, Oman, Kuwait, Bahrain

## Abstract

**Background:**

The United Nations considers children a crucial national asset and makes their welfare a top priority. However, infant mortality remains a persistent challenge, notably in Arab nations. Bahrain, Kuwait, and Oman, despite sharing similar income brackets and health care systems, differ in health policies, demographics, and maternal-child resource allocation. These countries also faced sharp fiscal deficits during the 2020 COVID-19 crisis. Compared to wealthier nearby nations like the United Arab Emirates, their lower gross domestic product further complicates efforts to reduce the Infant Mortality Rate (IMR) and sustain effective, equitable child health strategies.

**Objective:**

This study aimed to identify factors contributing to the IMR in Bahrain, Kuwait, and Oman by establishing an interpretative framework to examine the influence of sociodemographic, macroeconomic, health status and resource, and environmental factors.

**Methods:**

A longitudinal study collected annual time-series data (1990‐2022) for Bahrain, Kuwait, and Oman from international open sources. To counterbalance the time-series effects on both IMR and explanatory factors, a generalized least squares model based on the Cochrane-Orcutt procedure with a first-order autoregressive model was used.

**Results:**

Generalized least squares shows that the total fertility rate has a strong effect on IMR among the 3 countries (Oman: *β*=1.138, *P*<.001; Kuwait: *β*=.429, *P*=.006; Bahrain: *β*=.610, *P*=.03). Health status and resources, such as female life expectancy at birth, had an inconsistent impact on the IMR, with a positive effect (*β*=.103, *P*=.002) for Oman and a negative effect (*β*=−4.0697, *P*<.001) for Kuwait. Macroeconomic factors, such as female unemployment, were significant in decreasing the IMR only for Kuwait (*β*=−.076, *P*=.008). Gross domestic product per capita is significant only for Bahrain (*β*=−.398, *P*<.001). Environmental factors included CO_2_ emissions, which negatively impacted Oman’s IMR (*β*=−.077, *P*=.03), and N_2_O had a positive effect on Bahrain’s IMR (*β*=.420, *P*=.04).

**Conclusions:**

This study indicated the substantial effects of sociodemographics, health status and resources, macroeconomics, and environment on the IMR in 3 Arab countries. Sociodemographic and health-related factors like female life expectancy, fertility regulation, and female unemployment level were identified as key determinants of infant mortality.

## Introduction

### Background

Infancy is a crucial period in human life, the time of equal or greater dramatic developmental changes than any other in the human lifespan [1]. The most notable ones include capabilities, the complexity of the nervous system, the emergence of sensory and perceptual abilities, the development of communication skills, and many other factors. Therefore, most societies view children as a national asset and allocate a significant portion of their resources to their care and support. However, despite the improvements in health care, the Infant Mortality Rate (IMR) is still a problem in many regions of the world, especially in Arab countries [2]. The World Health Organization (WHO) defines IMR as “the probability of a child born in a specific year or period dying before reaching the age of one, if subject to age-specific mortality rates of that period” [3]. IMR provides an overview of the background conditions that the child lives in, and it is calculated using the annual number of infant deaths during a year in the numerator, divided by the total annual number of live births in the same year, multiplied by 1000, for the same geographic area and time [4]. According to data from the World Bank, Oman’s IMR in 1963 was drastically high, with 219 infant deaths per 1000 live births. The IMR, however, dropped to 9 infant deaths per 1000 live births in 2021 [5]. The same pattern applies to Bahrain; the IMR in 2021 was 5.9 deaths per 1000 live births, compared to 104 deaths in 1963. The declining trend in the IMR for Kuwait is evident, from 99 infant deaths per 1000 live births in 1963 to 8 deaths per 1000 live births in 2021. These 3 Gulf Cooperation Council (GCC) countries exhibit comparable IMR patterns and follow the anticipated worldwide historical decline.

A previous study determined the impact of various health resource determinants on IMR in Oman, considering that total health expenditure has a detrimental and statistically significant effect on IMR [2]. Another important aspect is ensuring that the mother has optimal health during the peripartum period, which substantially impacts the IMR. Mothers with multiple pregnancies, a history of infant mortality, or previous preterm births are associated with higher rates of infant death [6,7]. The leading cause of infant mortality in developing countries is infections, particularly infectious diseases that result in high neonatal mortality [8]. Infant health status also seems to have a significant impact on death rates. Preterm delivery and a low birth weight baby appear to be some of the most prominent causes of increased mortality [7].

Multiple studies have affirmed the relationship of some sociodemographic factors with the likelihood of infant deaths. For instance, a study showed that consanguinity indirectly increases the likelihood of congenital abnormalities, which may increase the risk of death from complications in infants born with these congenital malformations [9]. In addition, a study emphasized the need for implementing a national neonatal screening program to assess the exact burden of high rates of inborn errors of metabolism in Bahrain, aiming to reduce mortality and morbidity through early management [10].

Macroeconomic factors are significant predictors of IMR [2,11]. The countries in the Arabian Gulf displayed a special interest in regional child mortality indicators under the guidance of the Gulf Health Council. Studies reported that the Gulf countries have significantly improved the general health of their communities and significantly decreased IMR compared to the past as a result of the significant expansion of their economies and their performance as measured by their gross domestic product (GDP), both overall and per capita [11] and the same idea presented by another study [2].

The relationship between environment and national per capita income is clear [12]. Moreover, in some nations, there is a correlation between health and income, as they heavily rely on oil and gas exports. Oman, Kuwait, and Bahrain are known for having similar per capita income levels in the GCC countries. However, the widespread burning of fossil fuels over time has a detrimental effect on environmental quality. The primary greenhouse gases responsible for climate change are carbon dioxide (CO_2_), nitrous oxide (N_2_O), and methane. GCC states are major sources of CO_2_ emissions, a significant GHG component. Energy production, particularly the burning of fossil fuels (eg, coal, oil, and gas), and the region’s high energy consumption are factors in the increase in CO_2_.

Bahrain, Kuwait, and Oman are Arab countries that have the lowest GDP among other GCC members [13]. This suggests that fluctuations in economic productivity could be shaping infant mortality trends. Both Oman and Bahrain reflect comparatively fragile economic conditions for their populations, as evidenced by lower per capita foreign investment and modest central bank reserves [14]. Such indicators suggest a decline in financial resilience, which may limit the ability of expectant mothers to afford essential health care services, thereby affecting both fertility patterns and infant survival rates. Compounding this issue, women in many developing nations often shoulder substantial fiscal hardship, and the resulting gaps in perinatal care access are strongly linked to elevated infant mortality risks [8,15]. This means that their government spending exceeded their revenues, which could lead to debt and inflation problems that affect health care expenditure. Their total fertility rates have declined similarly, from an average of 7.2 in 1960 to 2.8 in 2020, comparable to those of other non-oil Arab countries following the Arab Spring [16]. This indicates that their population growth and structure have undergone significant changes over time, which could impact their social and economic development. In addition, the 3 Arab countries have had almost identical declining rates of infant deaths in the past 30 years. Furthermore, their research performance is the lowest in the GCC region, with a similar number of research articles per million population over the past 25 years [17]. While these countries exhibit similarities in income levels and health care infrastructure, they differ in specific health policies, population demographics, and resource allocations, particularly in relation to maternal and child health. For example, Oman and Bahrain emphasize preventive health care through vaccination programs and public health campaigns in allocating health resources. Kuwait has historically prioritized curative services in its health care strategy [12,14]. In terms of policy structure and vision, Bahrain’s health care strategy is increasingly aligned with its economic vision to enhance public health outcomes and support sustainable development, often leveraging international collaborations [15]. Oman’s policies focus on expanding access to rural health care, aligning with its commitment to decentralization [2,18]. In contrast, Kuwait’s policies focus on advanced medical facilities and specialist care, reflecting a more urban-centered approach [11,19]. In terms of population growth, Kuwait has experienced steady growth, driven primarily by expatriate labor demands. Oman, on the other hand, has a slower, more balanced growth between locals and expatriates, while Bahrain’s growth is urban-centric due to its smaller size [20]. By conducting this comparative analysis, this study aims to uncover unique insights into how these factors influence IMR among the 3 countries, and further research is needed to fill the existing gap in the literature.

### Purpose of the Study

This study aimed to identify factors contributing to the IMR in Bahrain, Kuwait, and Oman by establishing an interpretative framework to examine the influence of sociodemographic, health status and resources, macroeconomic, and environmental factors.

## Methods

### Study Design

For 33 years, from 1990 to 2022, this retrospective time-series study gathered data for Bahrain, Kuwait, and Oman. The time frame for the study was selected after the 1990s because these countries did not have access to accurate, official yearly data before that time.

### Data Resources

This study gathers yearly secondary data for Oman, Kuwait, and Bahrain. The data and descriptions were collected from the World Bank [5] and the Organization of Islamic Cooperation Statistics Database, which is maintained by the Statistical, Economic, and Social Research and Training Center for Islamic Countries [21].

### Ethical Considerations

Ethical approval was obtained from Sultan Qaboos University Medical Research Ethics Committee (#2666). This study used secondary data that did not involve any interaction with individual human participants and presented no reidentification risk due to the aggregated nature.

### Sample Size

This study’s sample size (number of years) was specified according to GLS with a first-order autoregressive model and using R software (“nlme” and “MASS” library) by the R Foundation. Suppose we expect the slope of the fertility rate on IMR to be 0.6 and the rho to be 0.5 in a first-order autoregressive model. At least, this study required a sample size of 30 years for each country, with approximately 71% power at a 5% type I error.

### Data Collection

We follow the evaluation criteria to ensure the degree of relevance and viability of these data [2]. A data cleaning process was also carried out, which was considered one of the key actions taken to ensure the quality of the data. Initially, the team collected data on health status and resources, sociodemographics, macroeconomics, and environment for each country based on available databases from the World Bank [5] and the Statistical, Economic, and Social Research and Training Center for Islamic Countries [21]. For sociodemographic, Bahrain and Kuwait included 10 variables: maternal or paternal educational level, maternal age, parental occupation, consanguinity, total fertility rate (births per woman), infant gender, birth rate, population density, and monthly income. For environmental, all 3 countries had data on 3 variables: CO_2_ (metric tons per capita), methane, and N_2_O emissions. In macroeconomics, Oman included 3 variables: GDP, GDP per capita, and female unemployment, while Bahrain and Kuwait included 6 variables: GDP, GDP per capita, gross national income, gross national income per capita, female unemployment, and inflation. For health status and resources, Oman and Bahrain included 13 variables: total health care expenditure, prematurity, birth weight, fetal growth, congenital abnormalities, neonatal sepsis, multiple pregnancies, history of preterm birth, history of infant death, delivery room resuscitation, mode of delivery, female life expectancy at birth, and immunization. Kuwait included the same 13 variables plus 5 additional variables: skilled health staff, access to safe water, hospital beds, and the number of doctors and nurses. Furthermore, any variable in this study that did not have data for one of the 3 countries was excluded because it cannot be used to compare across the 3 countries. Those independent variables with high multicollinearity with other variables will also be removed. In addition, variables with 20% or more missing data were eliminated and cannot be included in the analytical process. These variables require imputation, which is only imputed for 2 to 3 years, especially for CO_2_ and N_2_O in the environmental data for all 3 countries. Multiple regression imputation was used to fill in the missing data gaps for further analysis.

A recent systematic review and meta-analysis in GCC countries [19] provided the foundation for selecting relevant health status and resource, sociodemographic, and macroeconomic variables that affect IMR in the target countries. In addition, variables related to environmental factors were included based on other related studies [12]. Our investigation encompasses diverse independent variables spanning four key categories: macroeconomic, sociodemographic, health status and resources, and environmental. These variables collectively influence IMR, which serves as our research’s dependent variable and primary outcome. For further details, refer to Multimedia Appendix 1, which lists the relevant variables.

### Statistical Analysis

Descriptive statistics (eg, mean, SD, maximum, and minimum) were used to explore the characteristics of both IMR and explanatory variables for each target country. First, the variables within each factor, as outlined in the Data collection section, were identified as statistically significant factors of IMR across all 3 countries through univariate analysis. These variables, at *P*<.05, were subsequently integrated into a multivariable analysis for each country. To counterbalance the time-series effects on both IMR and explanatory factors, this study used generalized least squares (GLS) regression analysis based on the Cochrane-Orcutt procedure, which controls for a first-order autoregressive model. Other researchers have recommended this approach for analyzing national panels with time-series features [22-24]. By using GLS analysis, we can address our research objectives. In addition, this study assessed correlation (*r*≥0.8) and collinearity (variance inflation factor≥10) to identify any strong correlations among macroeconomic, sociodemographic, health status and resources, and environmental factors. Variables exhibiting strong correlations were excluded from the analysis [25]. All analyses were conducted using IBM SPSS and EViews 11, with a significance level set at 5%.

## Results

### Descriptive Characteristics of the Variables in Bahrain, Kuwait, and Oman

**Figure 1.**
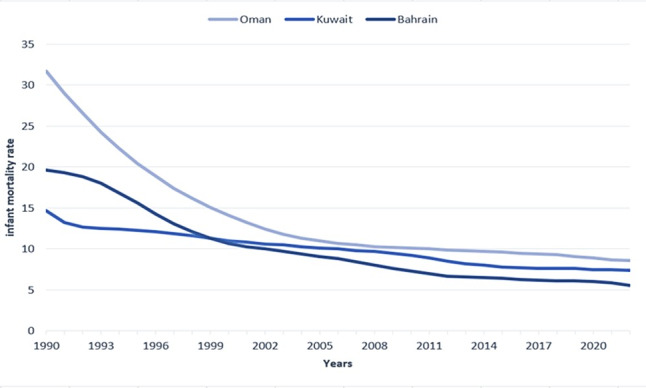
Infant mortality rate in Oman, Kuwait, and Bahrain (1990‐2022).

This study compiled 33 years of macroeconomic, sociodemographic, health status, and resource, environmental, and IMR data from 1990 to 2022 in three Arab countries (refer to Table 1). Oman significantly reduced IMR, dropping from 31.7 to 6.4 deaths per 1000 live births. Bahrain followed with a decrease from 19.6 to 5.3, and Kuwait experienced a reduction from 14.7 to 6.3 deaths per 1000 live births (refer to Figure 1). However, the average IMR was 10 deaths per 1000 live births in Kuwait and Bahrain, while Oman had a slightly higher average of nearly 14 deaths per 1000 live births. In terms of total fertility rate, Oman had the highest rate at 3.7 births per woman, while Bahrain had the lowest at 2.5 births per woman. When considering female life expectancy at birth, Kuwait had the highest at 79.7 years, and Bahrain had the lowest at 75.8 years. In terms of economics, Oman had the lowest GDP per capita at US $14,056.8, while Kuwait had the highest at US $28,678.9. Regarding CO_2_ emissions, Kuwait had the highest level at 23.5 metric tons per capita, while Oman had the lowest at 12.8 metric tons per capita.

**Table 1.** Descriptive statistics of variables of Oman, Kuwait, and Bahrain (1990‐2022). Each variable was transformed into natural logarithms in the analysis.

Country	IMR^a^	TFR^b^	Female LE^c^ at birth	GDP^d^ per capita (US $)	Female unemployment	CO_2_^e^ emissions	N_2_O^f^ emissions
Oman							
Mean (SD)	13.9 (6.5)	3.7 (1.2)	76.9 (2.1)	14056.8 (6971.9)	3.7 (0.7)	12.8 (3.5)	652.9 (239.5)
Range	6.4‐31.7	2.6‐6.6	72.3‐80.0	5984.4‐25056.8	1.8‐4.3	6.6‐17.1	310.7‐1064.7
Kuwait							
Mean (SD)	10.0 (2.1)	2.6 (0.4)	79.7 (1.7)	28678.9 (13366.9)	3.5 (1.9)	23.5 (4.9)	619.8 (238.8)
Range	6.3‐14.7	2.1‐3.3	76.8‐82.8	8219.5‐55595.4	1.5‐7.8	5.4‐31.3	169.6‐1003.6
Bahrain							
Mean (SD)	10.1 (4.4)	2.5 (0.6)	75.8 (2.4)	17655.4 (7062.4)	3.8 (0.2)	22.1 (0.8)	101.7 (41.5)
Range	5.3‐19.6	1.2‐3.8	71.8‐79.0	8174.8‐30152.0	3.6‐4.7	19.3‐23.2	50.3‐181.3

a IMR: infant mortality rate.

b TFR: total fertility rate.

c LE: life expectancy.

d GDP: gross domestic product.

e CO_2_

f N_2_O

### Associated Factors on IMR for Each Country

In the univariable analysis, the total fertility rate and female life expectancy at birth were significant (*P*<.05) across all sociodemographic and health status and resource variables in all 3 countries. In macroeconomics, female unemployment and GDP per capita (in USD) were significant (*P*<.05) in all countries. In addition, CO_2_ and N_2_O emissions in environmental factors were also found to be significant (*P*<.05) across all countries. These 6 variables were included in the GLS regression model to explore which factors were associated with IMR in each country. Table 2 displays the explanatory factors associated with IMR for each country, showing significance in the model. The analysis reveals that the total fertility rate has a strong effect on IMR among the three countries: Oman (*β*=1.138, *t*=8.988, *P*<.001); Kuwait (*β*=.429, *t*=2.965, *P*=.006); Bahrain (*β*=610, *t*=2.310, *P*=.03). However, the study shows that female life expectancy at birth had an inconsistent impact on the IMR. It had a positive effect on IMR for Oman (*β*=.103, *t*=3.541, *P*=.002) but a negative effect on IMR for Kuwait (*β*=−4.069, *t*=5.588, *P*<.001), while having no significant effect (*β*=.137, *t*=0.998, *P*=.33) on IMR for Bahrain. In addition, the macroeconomic factors represented female unemployment and GDP per capita. Female unemployment was significantly associated with IMR only in Kuwait (*β*=−.076, *t*=2.868, *P*=.008), and GDP per capita was significant only for Bahrain, with a β coefficient of −.398 (*t*=−3.854, *P*<.001). Moreover, the model indicates a relationship between CO_2_ and N_2_O emissions and IMR; specifically, CO_2_ emissions have a negative impact on Oman’s IMR (*β*=−.077, *t*=2.370, *P*=.03). However, N_2_O emissions have a positive and direct effect on the IMR in Bahrain (*β*=.420; *t*=2.118, *P*=.044).

**Table 2.** Comparison of the effect of sociodemographic, health status and resources, macroeconomic, and environmental factors (1990‐2022) on IMR^a^ in Oman, Kuwait, and Bahrain by GLS^b^ regression model.

	Oman			Kuwait			Bahrain		
Factor	β (SE)^c^	t statistics^d^	*P* value	β (SE)	t statistics	*P* value	β (SE)	t statistics	*P* value
Sociodemographic									
Total fertility rate	1.138 (.126)	8.988	<.001	.429 (.145)	2.965	.006	.610 (.264)	2.310	.03
Health status and resources									
Female life expectancy at birth	.103 (0.029)	3.541	.002	−4.069 (.728)	-5.588	<.001	.137 (.138)	.998	.33
Macroeconomic									
Female unemployment	−.122 (.059)	−2.044	.053	−0.076 (.026)	−2.868	.008	.489 (.365)	1.341	.19
GDP per capita (US $)	−.057 (.054)	−1.053	.30	−.001 (.031)	−.038	.97	−.398 (.103)	−3.854	<.001
Environmental									
CO_2_ emissions	−.077 (.032)	−2.370	.03	.015 (.016)	.913	.37	.077 (.068)	1.127	.27
N_2_O emissions	−.232 (.195)	−1.190	.25	−.003 (.008)	−.350	.73	.420 (.198)	2.118	.044

a IMR: Infant mortality rate.

b GLS: Generalized least squares.

c SE: standard error.

d Statistics: *t*-statistics.

## Discussion

### Principal Findings

This study used GLS with a first-order autoregressive model to investigate the determinants influencing the IMR in Oman, Bahrain, and Kuwait. These determinants, including fertility, female life expectancy, unemployment, GDP per capita, and environmental pollutants, intertwine uniquely across 3 Arab countries, shaping infant mortality patterns. These contextual determinants highlight how sociodemographic, health resources, macroeconomic, and environmental forces converge to influence newborn survival outcomes in complex, country-specific ways.

### The Effects of Sociodemographic Factors on IMR

Among the 3 countries, the sociodemographic determinant, represented by the total fertility rate, has a direct and strong effect on IMR, consistent with the findings of other studies. Internationally, the impact of maternal education on infant mortality may be mediated by fertility [26,27]. Regionally, numerous studies conducted in Oman have examined the impact of various factors on the IMR. Their findings showed that the fertility rate significantly predicts IMR [2,18]. Our study showed that the total fertility rate in Oman is the highest (b=1.138, *P*<.001), followed by Bahrain (b=0.610, *P*=.029) and Kuwait (b=0.429, *P*=.006) in terms of IMR in their respective countries. The total fertility rate represents the number of children that would be born to a woman if she were to live to the end of her childbearing years. A greater probability of complications developing during pregnancy is associated with a higher fertility rate, which implies that the mother will give birth more frequently [19], and any pregnancy complications may increase the risk of mortality for both the infant and the mother [28].

### The Effects of Health Status and Resource Factors on IMR

In Oman, the female life expectancy at birth has a direct impact on IMR (*β*=.103, *P*=.002). However, it has a negative impact on IMR in Kuwait (*β*=−4.069, *P*<.001), and it is not significant in Bahrain (b=0.137, *P*=.33). This indicates the number of years a female newborn would survive if the mortality patterns at the time of the birth continued to remain the same throughout her life [11]. Oman’s first documented IMR was in 1963, which was drastically high. However, this rate dramatically declined across the years until it reached 9 baby deaths per 1000 live births in 2021. Indeed, it was a significant decline, but more needs to be done to maintain it. Since Oman’s IMR is still the highest in the GCC despite similar economic conditions, indicating that if the health policies, medical facilities, cultural norms, and economic status, therefore, the mortality patterns that were in place at the time of the female’s birth continued to remain the same or worse, the IMR will increase. However, Kuwait’s IMR is slightly better, reaching 8 deaths per 1000 live births in 2021. If the mortality patterns and factors associated with them that were in place at the time of the female’s birth continued to remain the same or improved, this would further decrease the IMR. In contrast, the lack of a significant relationship between female life expectancy at birth and IMR in Bahrain may be due to a more direct impact on infant death rather than female life expectancy. Our findings suggest that the IMR in Bahrain is influenced by macroeconomic factors, specifically GDP per capita, as well as environmental factors, particularly N_2_O emissions. This pattern differs from the other 2 countries analyzed, where these variables showed varying degrees of impact. These findings suggest that specific factors, such as economic structures and environmental conditions, play a critical role in infant death in Bahrain.

### The Effects of Microeconomic Factors on IMR

This study found that female unemployment has a negative effect on the IMR in Kuwait (b=−0.076, *P*=.0008), while it is not significant in Oman (b=−0.122, *P*=.05) and Bahrain (b=0.489, *P*=.19). This finding was consistent with a study done for Macau [29]. However, numerous studies have emphasized the critical significance of a higher proportion of the employed population in lowering the IMR regionally [18] and internationally [30]. In addition, this result contrasts with the GCC region systematic review, which concluded that only paternal working status matters and that maternal employment had no impact on IMR [19]. Being a housewife is associated with providing better care, spending more quality time with the baby, and meeting the child’s psychological and physical needs. As a possibility, society has left the child’s responsibility and care to the mother alone, and the father has no connection to the matter. Therefore, if the mother pays little attention to her work, the child will remain without care, which leads to a high infant mortality rate. We recommend further research to investigate why female unemployment has a negative impact on the IMR in Kuwait. Additionally, our findings showed that GDP per capita significantly reduced the IMR only in Bahrain (b=−0.398, *P*<.001) but not in Oman (b=−0.057, *P*=.302) or Kuwait (b=−0.0011, *P*=.97). A study in Oman reported a contrasting negative impact of GDP per capita on IMR [2]. This difference may stem from our inclusion of a broader data range and environmental factors such as CO_2_ emissions, which exert a more direct influence on IMR in Oman than GDP per capita. Furthermore, the lack of a significant relationship between GDP per capita and IMR in Kuwait could be attributed to the multifactorial nature of health care outcomes in the country. GDP per capita likely impacts healthcare quality and equity rather than directly determining IMR. Other determinants, such as the total fertility rate, female life expectancy at birth, and female unemployment, may dilute the direct effects of GDP per capita on IMR. These findings underscore the importance of context-specific analyses in understanding the intricate relationship between economic indicators and health outcomes.

### The Effects of Environmental Factors on IMR

This study has indicated that environmental determinants represented by CO_2_ emissions negatively impact Oman’s IMR (*β*=−.077, *P*=.026). A study found that CO_2_ emissions and nonrenewable energy are indirectly essential determinants of the IMR [31]. They have varying effects on the different income groups. This implies that nonrenewable energy indirectly increases the mortality rate for people in lower-middle and upper-middle-income countries but reduces it in high-income countries [31]. As indicated earlier, Oman lacks the high-income capacity that would enable it to be a country with higher CO_2_ emissions control, so we recommend further studies based on this idea. High nonrenewable energy use can be related to the country’s better economic and employment status, so it depends on its ability to control CO_2_ emissions. However, previous studies have demonstrated that respiratory disorders and a high mortality rate are primarily caused by environmental pollution. Moreover, the model indicated that the environmental construct in Bahrain is best reflected by N_2_O emissions (*β*=.420, *P*=.044). N_2_O emissions contribute to the global accumulation of greenhouse gases, leading to adverse environmental and health effects. Numerous studies have documented the health risks associated with nitrate toxicity, including methemoglobinemia in newborns and its strong correlation with cancer [32].

### Limitations and Future Directions

Many variables considered had potential significance according to the previous studies. However, with incomplete data, many variables were excluded from the analysis. Nevertheless, these variables may still affect the IMR in each country, so interpreting the results requires caution due to this issue. Furthermore, more studies using longer longitudinal data are recommended. Another limitation of this research is related to the analysis method. The GLS model does not account for the indirect effects of explanatory variables on IMR. More studies should be recommended to examine its impact on IMR among countries.

### Conclusions

This analysis revealed the substantial impact of sociodemographic, health status, and resource factors, as well as macroeconomic and environmental factors, on the IMR in 3 Arab countries. Sociodemographic and health-related factors like female life expectancy, fertility regulation, and female unemployment level were identified as key determinants of infant mortality. Based on the empirical facts regarding the environment, promoting clean energy development to enhance human health is recommended. In other words, the IMRs of these countries are expected to decline even further due to the adoption of environmental quality policies. The results of this study suggest improvements and interventions that consider macroeconomic, environmental, sociodemographic, and health status-related issues.

## Supplementary material

10.2196/73203 Multimedia Appendix 1 Lists of factors and variables used in the study for each country.

## Abbreviations

CO_2_: carbon dioxide
GCC: Gulf Cooperation Council
GDP: gross domestic product
GLS: generalized least squares
IMR: infant mortality rate
N_2_O: nitrous oxide
WHO: World Health Organization
