# Supplementary material for: Comparing Sociodemographic, Health Status and Resources, Macroeconomic Status, and Environmental Factors on Infant Mortality Rates in Bahrain, Kuwait, and Oman: Longitudinal Time-Series Study
Source: JMIR Pediatr Parent. 2025 Oct 10;8:e73203. doi: 10.2196/73203 (PMC12513711; doi:10.2196/73203)
Supplement: Multimedia Appendix 1 [file pediatrics-v8-e73203-s001.docx]

**Multimedia Appendix 1**

| **Table S1. Lists of factors and variables used in the study for each country.** | | | |
| --- | --- | --- | --- |
| **Factor** | **Variable** | **Definition** | **Source** |
| IMR | Infant mortality rate | The number of infants dying before reaching one year of age per 1,000 live births in a given year. | World Bank [5],  SESRIC [21] |
| SD | Total fertility rate (total births per woman) | The total fertility rate represents the number of children born to a woman if she lived to the end of her childbearing years and bore children following age-specific fertility rates of the specified year. | World Bank [5] |
| HSR | Female life Expectancy at birth | The number of years a newborn infant would live if prevailing patterns of mortality at the time of its birth were to stay the same throughout its life. | SESRIC [21] |
| ME | Unemployment, female (% of female labor force) | The proportion of a country’s unemployed female population is expressed as a percentage. | World Bank [5] |
|  | GDP per capita (current USD) | GDP per capita is the gross domestic product (GDP) divided by the mid-year population. | World Bank [5] |
| EN | Carbon dioxide emissions (metric tons per capita) | CO_2_ emissions are those stemming from the burning of fossil fuels and the manufacture of cement. They include carbon dioxide produced during the consumption of solid, liquid, and gaseous fuels, as well as gas flaring. | World Bank [5] |
|  | Nitrous oxide emissions | N_2_O emissions are from agricultural biomass burning, industrial activities, and livestock management. | World Bank [5] |
| IMR, Infant mortality rate; SD, sociodemographic factors; HSR, Health service and resources; ME, microeconomic; EN, Environmental; SESRIC, Statistical, Economic and Social Research and Training Centre for Islamic Countries. | | | |
